# Quantification of Anterior Chamber Particles Using Anterior Segment Optical Coherence Tomography in Angle-Closure Glaucoma Patients after Laser Iridotomy

**DOI:** 10.3390/jcm11154379

**Published:** 2022-07-28

**Authors:** Naoya Yoshihara, Hiroto Terasaki, Hideki Shiihara, Ryoh Funatsu, Takehiro Yamashita, Taiji Sakamoto

**Affiliations:** Department of Ophthalmology, Kagoshima University Graduate School of Medical and Dental Sciences, Kagoshima 890-8520, Japan; yoshihara_eye@yahoo.co.jp (N.Y.); 58149@m2.kufm.kagoshima-u.ac.jp (H.S.); k7890207@kadai.jp (R.F.); take1@po4.synapse.ne.jp (T.Y.); tsakamot@m3.kufm.kagoshima-u.ac.jp (T.S.)

**Keywords:** anterior segment optical coherence tomography, anterior chamber, objective quantification, laser iridotomy

## Abstract

Purpose: To determine whether the degree of particle density in the anterior chamber can be evaluated objectively and quantitatively by anterior segment optical coherence tomography (AS-OCT) in cases after laser iridotomy (LI). Methods: This was a retrospective observational study. All of the subjects who received LI for angle-closure glaucoma between January 2018 and May 2019 at Kagoshima University Hospital were studied. AS-OCT recordings were made before, immediately after, and one week after LI in 22 eyes of 14 consecutive patients. The anterior chamber particle (ACP) index was defined as the ratio of the number of particles in the anterior chamber to the total area of the anterior chamber. The ACP index was determined by binarization of the AS-OCT images and analysis with the ImageJ program. Results: The mean age of the participants was 75.4 ± 8.9 years, with a range of 61–91 years. The ACP index before the LI was 0.78 ± 0.68, and it was significantly increased to 7.72 ± 2.64 immediately after the LI (paired *t*-test, *p* < 0.01). The ACP index returned to the pre-LI density of 0.92 ± 0.48 one week after the LI. Conclusions: We successfully quantified the degree of anterior chamber particles accumulation by analyzing images obtained by AS-OCT. This simple and repeatable technique should be useful because the particles, including inflammatory cells, in the anterior chamber can be evaluated non-invasively and objectively.

## 1. Introduction

An evaluation of the pathological changes in the anterior chamber of the eye is important for the diagnosis of many eye diseases, and it is an essential test in daily clinical practice. Slit-lamp biomicroscopy is most frequently used for this evaluation [1,2]. As this method is subjective, the results may not be repeatable or reproducible among examiners and even the same examiner on different days. There is also a laser flare-cell meter that can perform an objective evaluation of the degree of inflammation in the anterior chamber by quantifying the concentration of protein in the anterior chamber as a flare value. It can also count the number of cells in the anterior chamber [3,4]. However, this instrument can only evaluate the degree of protein concentration and cannot measure the morphology of the anterior chamber. Thus, the flare meter may be useful for research purposes, but it is not useful for daily clinical practice.

**Figure 1:**
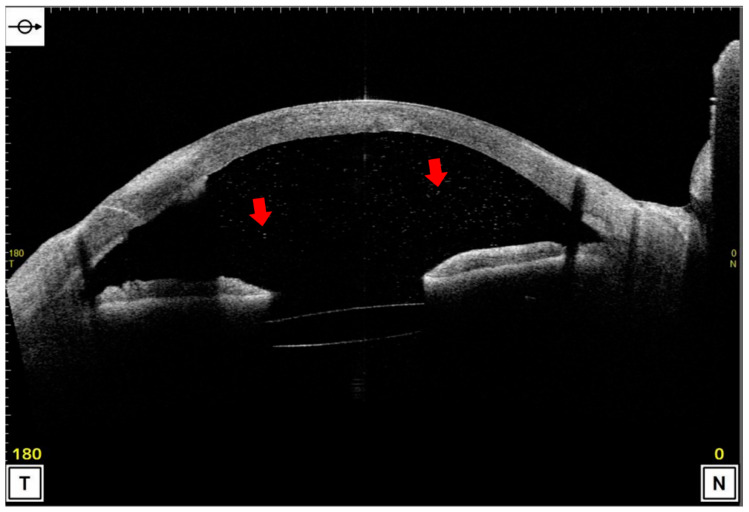
Case with particles in the anterior chamber. The finding in a case with particulate materials in the anterior chamber detected by ophthalmoscopy due to a corneal infection. When the anterior segment optical coherence tomographic (AS-OCT) images were examined, many particles considered to be inflammatory cells were found in the anterior chamber (indicated by arrows).

Anterior segment optical coherence tomography (AS-OCT) was recently made available for everyday clinical use, which has made it possible to acquire clear images of the anterior segment of the eye non-invasively. This device can evaluate the size, shape, and contents of the anterior chamber objectively and accurately, e.g., in eyes with primary angle-closure glaucoma or with a filtration bleb after trabeculectomy [5,6]. It is also possible to detect and quantify fine particulate materials in the anterior chamber in AS-OCT images (Figure 1).

The results of earlier studies showed that the particulate opacities in the anterior chamber in AS-OCT images can be inflammatory cells [7,8]. Several studies have reported quantifying cells in the anterior chamber using AS-OCT images [9,10]. These methods involve software that automatically counts anterior chamber cells [9] and counting cells manually using ImageJ software [10]. These are useful methods that correlate with the evaluation of inflammation using slit-lamp findings and have attracted attention, especially in the field of uveitis. On the other hand, software that automatically counts the number of cells in the anterior chamber is unavailable at all institutions. Manual counting can also be difficult when large numbers of red blood cells are present in the anterior chamber after glaucoma surgery or a vitrectomy for vitreous hemorrhage. In addition, the area of the anterior chamber changes after glaucoma surgery, laser iridotomy, cataract surgery, and vitrectomy. Thus, there are cases where it is difficult to evaluate changes in the anterior chamber only by cell counting. Therefore, we developed a method to assess inflammation in the anterior chamber by dividing a high signal in the anterior chamber, which corresponds to cells and pigments, by the anterior chamber area in the AS-OCT.

It is well known that the degree of inflammation and bleeding and the number of iris pigment granules in the anterior chamber increase immediately after laser iridotomy (LI) [11,12]. At that time, numerous pigmented and inflammatory cells and blood cells from the iris are present in the anterior chamber of the eye [11,12]. These changes in the anterior chamber before and after LI can be qualitatively determined by slit-lamp biomicroscopy. This should, then, be a good model to use to determine whether AS-OCT can obtain a quantitative value of the density of particulate materials in the anterior chamber by our method.

Thus, the purpose of this study was to determine whether AS-OCT can be used to quantify the density of particulate materials in the anterior chamber of the eye. To accomplish this, we recorded AS-OCT images of the anterior chamber (AC) before and after LI. The densities of the particulate matter in the AC were quantified by the binarization method, using the AS-OCT images and ImageJ software.

## 2. Materials and Methods

### 2.1. Ethics Statement

This was a retrospective study of 22 eyes of 14 consecutive patients who were scheduled to undergo LI therapy. The procedures used were approved by the Institutional Review Board of Kagoshima University Hospital, Kagoshima, Japan, and they conformed with the tenets of the Declaration of Helsinki. The Institutional Review Board also approved the collection and publication of the data from the medical charts of the patients.

### 2.2. Subjects and Examination Methods

Twenty-two eyes of 14 consecutive patients (5 men and 9 women) who were scheduled for LI for angle-closure glaucoma between January 2018 and May 2019 at the Kagoshima University Hospital were studied. Prior to the LI treatment, all of the eyes had a comprehensive ocular examination that included slit-lamp examinations of the anterior segment of the eye and ophthalmoscopic examinations of the fundus. The intraocular pressure (IOP) was measured with a pneumotonometer (CT-80, Topcon, Tokyo, Japan), and the axial length (AL) was measured with an AL-2000 ultrasound instrument (Tomey, Tokyo, Japan). Best-corrected visual acuity (BCVA) was measured after determining the refractive error with an Auto Kerato-Refractometer (RM8900, Topcon). All of the patients underwent swept-source Fourier-domain AS-OCT (CASIA2, Tomey Corporation, Nagoya, Japan) examinations before, immediately after, and 7 days after the LI treatment. In this study, a standardized 16-line radial scan was performed with AS-OCT and a horizontal scan was used for analysis. Each image was saved as a JPEG file with a size of 1414 × 1000 pixels.

### 2.3. Laser Iridectomy (LI)

LI was performed on all cases in accordance with the guidelines of the Japan Glaucoma Society [13]. Briefly, the first laser application was performed with a multicolor laser (Novus Varia, Lumenis, Dreieich, Germany), and the second application was performed with a neodymium-doped yttrium aluminum garnet (Nd:YAG) laser (Tango reflex, Ellex, Minneapolis, MN, USA). LI was performed by a single ophthalmologist (NY).

### 2.4. Evaluation of Anterior Chamber Inflammation by AS-OCT

**Figure 2:**
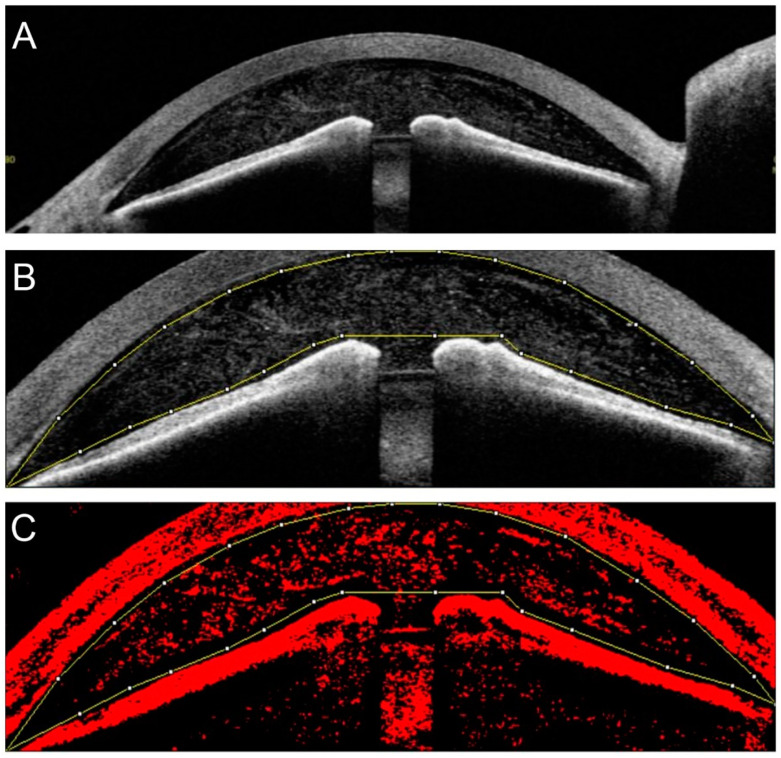
Quantification of anterior chamber particles. A horizontal image obtained by AS-OCT was imported into the ImageJ program (**A**). The interior of the anterior chamber was manually outlined as widely as possible with ImageJ (**B**). Then, the image was binarized using “bernsen” in “auto local threshold” mode, and the ratio of the particles in the anterior chamber to the total area of the anterior chamber was calculated (**C**).

AS-OCT was performed before and within 30 min after the LI. AS-OCT was also performed one week after the LI on 22 eyes. All images were obtained in the automatic mode, and a horizontal image of the anterior segment OCT was used for the analysis (Figure 2A).

### 2.5. Processing of AS-OCT Images

To quantify the densities of the particles in the anterior chamber objectively, the images of the anterior segment OCT were binarized with ImageJ software (version 1.47; The National Institutes of Health, Bethesda, MD; available at: http://imagej.nih.gov/ij/, accessed on 10 January 2020) [14]. Briefly, the interior of the anterior chamber was manually outlined as large as possible (Figure 2B), and this area was binarized using “bernsen” in the “auto local threshold” mode (Figure 2C). The total area of the anterior chamber and the total area of the particles in the anterior chamber were calculated. Then, the ratio of the area of the particles in the anterior chamber to the total area of the anterior chamber was calculated using the following formula and defined as the “anterior chamber particle (ACP) index”:

Anterior chamber particle (ACP) index = area of the signals from the particles/area of anterior chamber × 100 (%).

### 2.6. Intra-Examiner and Inter-Examiner Correlations

To determine the intra-examiner correlation, the same examiner (NY) examined the same set of images on two occasions with a one-week interval. To determine the inter-examiner correlation, measurements were performed by two examiners (NY, HS), and the ACP indices were compared.

### 2.7. Correlation of ACP Index with Other Ocular Parameters and Number of Laser Shots

Spearman’s rank correlation coefficient was used to determine the significance of the correlations between the ACP index immediately after the LI and the ocular parameters or the number of laser burns. The number of laser burns was determined from the medical records.

### 2.8. Statistical Analyses

All statistical analyses were performed with the statistical language R program (version 3.0.2, The R Foundation for Statistical Computing, Vienna, Austria). The significance of the difference in the ACP index before and after LI was analyzed by paired *t*-tests. The correlation between ACP index and each factor was analyzed using Spearman’s rank correlation coefficient. A *p*-value of <0.05 was taken to be statistically significant.

## 3. Results

### 3.1. Demographics of Patients

The mean age of the patients was 75.3 ± 8.9 years (±standard deviation) with a range of 61 to 91 years. The mean IOP was 12.8 ± 5.4 mmHg, with a range of 9 to 30 mmHg. The mean anterior camber depth before LI was 1.97 ± 0.35 mm, and it was 1.98 ± 0.33 mm immediately after the LI. The mean number of argon laser burns was 63.0 ± 28.8 and the mean number of YAG laser shots was 1.57 ± 1.4 (*p* > 0.05; Table 1).

### 3.2. Analysis of Cells in the Anterior Chamber before and after Laser Iridotomy

**Figure 3:**
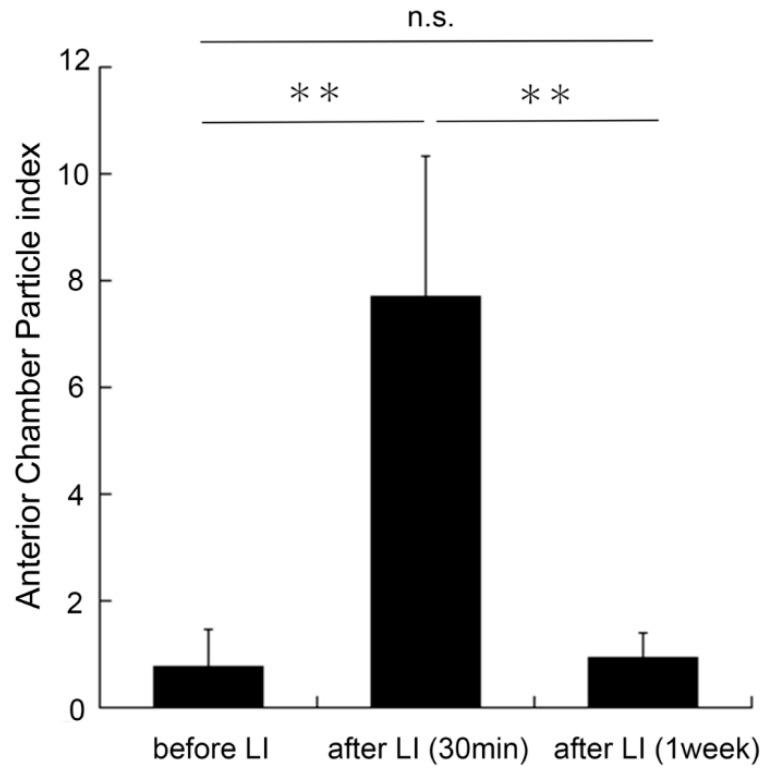
Anterior chamber particle (ACP) index before and after laser iridotomy (LI). ** *p* < 0.01.

A mean time of 3.18 ± 0.39 min was required to determine the ACP index from the AS-OCT images. The ACP index before the LI was 0.78 ± 0.68, and it increased significantly to 7.72 ± 2.64 immediately after the LI (*p* < 0.01; paired *t*-test). The ACP index then decreased to 0.92 ± 0.48 one week after the LI (*p* > 0.05 to baseline value; Figure 3).

The ACP index before the LI was 0.78 ± 0.68, and it increased significantly to 7.72 ± 2.64 after the LI (*p* < 0.01, paired *t*-test). One week after the LI, the ACP index decreased to 0.92 ± 0.48 (*n* = 9), which was not significantly different from the before-LI value.

### 3.3. Inter- and Intra-Rater Correlations of ACP Index Measurements and Correlations between Ocular Parameters and ACP Index

**Figure 4:**
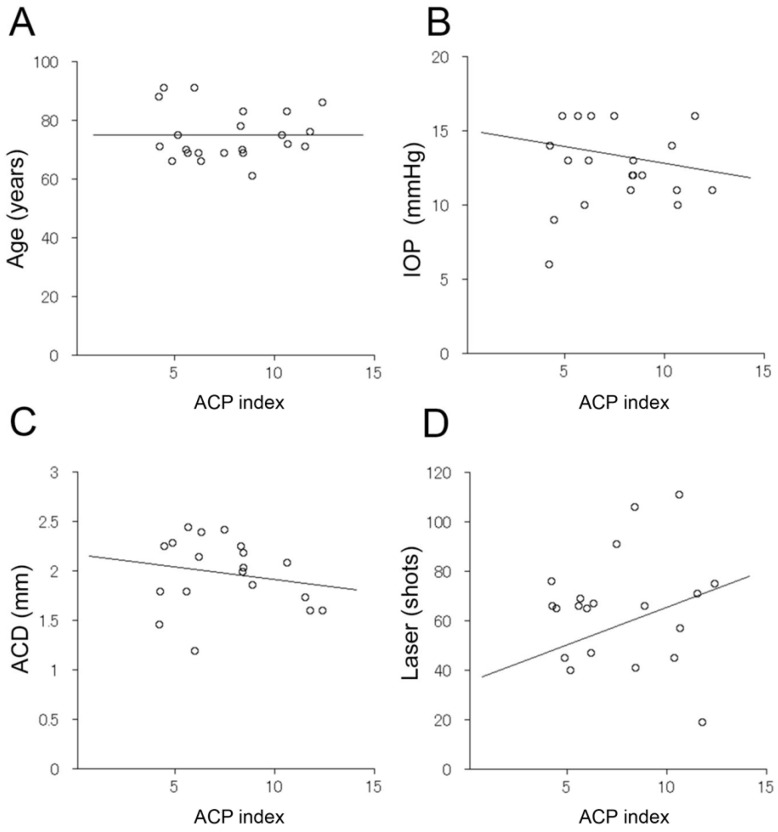
Correlations between ACP index and each factor. The correlations between ACP index immediately after LI and age, intraocular pressure, anterior chamber depth, and total number of lasers were determined. No significant correlations were observed (**A**, ACP index vs. age, r = 0.081 (*p* = 0.782); **B**, ACP index vs. IOP, r = −0.198 (*p* = 0.377); **C**, ACP index vs. ACD, r = −0.184 (*p* = 0.450); **D**, ACP index vs. total PC, r = 0.082 (*p* = 0.717)).

The intra-examiner correlation coefficient was 0.973, and the inter-examiner intra-class correlation coefficient was 0.996, indicating high reliability. The correlations between the ACP index immediately after the LI and age, intraocular pressure, anterior chamber depth, and the number of lasers (total number of multicolor lasers and Nd:YAG) were not significantly correlated (ACP index vs. age: r = 0.081 (*p* = 0.782), ACP index vs. IOP: r = −0.198 (*p* = 0.377), ACP index vs. ACD: r = −0.184 (*p* = 0.450), ACP index vs. total PC: r = 0.082 (*p* = 0.717)) (Figure 4).

### 3.4. Representative Case

**Figure 5:**
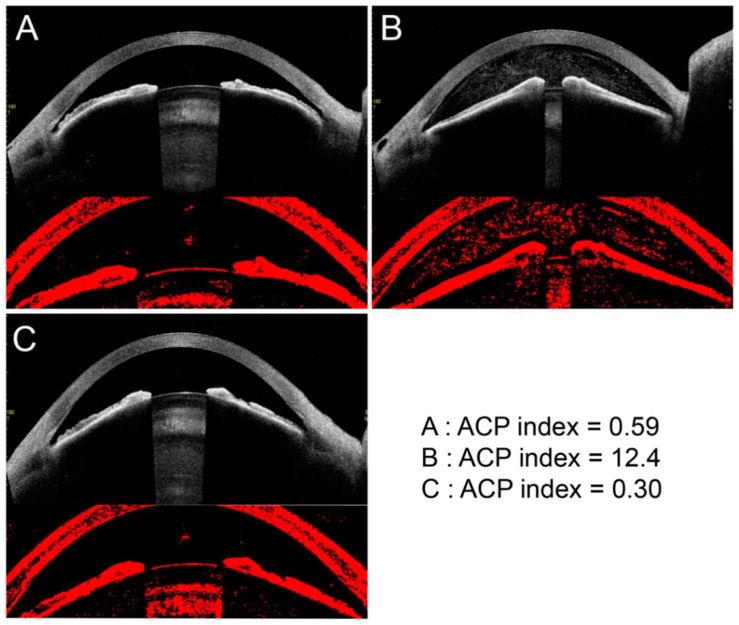
Representative case. Findings for an 86-year-old woman. There were no notable complications before and after LI. The ACP index increased and decreased in response to subjective changes in the anterior chamber inflammation. ACP index before LI = 0.59 (**A**), ACP index immediately after LI = 12.4 (**B**), and ACP index one week after LI = 0.30 (**C**).

The findings for an 86-year-old woman with no notable complications before and after LI are presented in Figure 5. The ACP index increased after the LI and then decreased one week after the LI. The ACP index before the LI was 0.59 (Figure 5A), immediately after the LI it was 12.4 (Figure 5B), and one week after the LI it was 0.30 (Figure 5C).

## 4. Discussion

Our results showed that it was possible to evaluate the density of the particulate material in the anterior chamber of the eye using images obtained by AS-OCT. Recently, several studies have reported quantifying cells in the anterior chamber using AS-OCT images [9,10]. These methods include software that automatically counts anterior chamber cells [9] and counting cells manually using ImageJ software [10]. These techniques correlate with subjective evaluation and are very useful in objectively assessing anterior segment inflammation with AS-OCT. However, as stated in the Introduction, there are cases where it is difficult to evaluate changes in the anterior chamber only by cell counting. Our findings concern the objective quantification of the “density” of particles in the anterior chamber. The determination of the ACP indices revealed very high intra- and inter-examiner correlation coefficients. Together, these findings indicate that AS-OCT findings are reliable and that they can be applied by investigators from different institutions and by the same clinicians in assessing the effects of treatments.

There are several advantages of using AS-OCT to determine the density of particles in the anterior chamber. First, because AS-OCT uses a long laser wavelength of 1310 μm, a clear tomographic image can be obtained even in cases with corneal opacities [7,15]. In such cases, it is difficult to evaluate findings in the anterior chamber by slit-lamp biomicroscopy. Another advantage is that it is easy to use and is non-invasive and can be performed repeatedly. Thus, this method should be helpful in assessing particles, including inflammatory cells, blood cells, and iris pigment granules, in the anterior chamber before and during the course of treatments. Another advantage of this technique is the high level of inter-examiner correlations, which allow the data obtained by two examiners to be compared without any drawbacks.

OCT research has been conducted mainly in relation to the treatment of retinal diseases, with the digitization of the images and using two levels of gradation [14,16,17,18,19]. The results of this study showed the efficacy of AS-OCT in investigating anterior segment diseases. However, the lack of significant correlations between the ACP index and BCVA acuity, intraocular pressure, total number of lasers, and anterior chamber depth was puzzling. However, it is highly likely that the particles in the anterior chamber may not only be inflammatory cells but also iris pigments and blood cells. These factors might have affected the results.

The total number of laser shots was also not significantly correlated with the ACP index. Since lasers coagulate tissues, a larger number of laser shots might not have induced the release of more particles into the anterior chamber. In addition, because the LIs were performed by an experienced ophthalmologist, the small variations in the number of laser applications may not have affected the densities of particles.

There are limitations to this study. First, all of the eyes were those of primary angle-closure glaucoma that were treated with LI. The particles in the anterior chamber might contain different types of cells, such as inflammatory cells, iris pigment cells, and blood cells. Thus, the ACP index is not totally indicative of the degree of inflammation in the anterior chamber. Classification of the particles would be helpful in the diagnosis of the disease and should be studied in the future. Second, we did not compare the semi-objective grading of inflammation in the anterior chamber, which is most frequently used in clinical settings with the ACP index. Semi-objective grading is expressed as 4+ to 0 based upon the graders’ impression. The degree of inflammation was expressed as 3+ or more in most of the cases. Thus, no meaningful correlation was obtained by the comparison. Our method is superior because the strength of inflammation can be expressed quantitatively, even in the case of eyes with strong inflammation.

In conclusion, quantification of anterior chamber particles using the ACP index will allow clinicians to make easy and objective evaluations of particles in the anterior chamber. As it is easy to perform and non-invasive and because of its high intra- and inter-examiner correlations, we recommend AS-OCT to quantify the degree of particle density in the anterior chamber of the eye. Objective methods for assessing intra-anterior chamber particles using AS-OCT have received attention mainly in the area of uveitis. However, they may also be helpful in the evaluation of such particles as inflammatory cells and blood cells in the anterior chamber after cataract or vitreous surgery, and thus require further investigation.

## Figures and Tables

**Table 1:** Demographics of patients.

	Average	Range
**Age (years)**	75.3 ± 8.9	61–91
**IOP (mmHg)**	12.8 ± 5.4	9–30
**ACD before LI (mm)**	1.97 ± 0.35	1.46–2.44
**ACD after LI (mm)**	1.98 ± 0.33	1.24–2.58
**Multicolor laser (shots)**	63.0 ± 28.8	18–130
**Nd:YAG laser (shots)**	1.57 ± 1.4	1–7

IOP, intraocular pressure; ACD, anterior chamber depth; LI, laser iridotomy; Nd:YAG, neodymium-doped yttrium aluminium garnet.

## Data Availability

Data are available from the corresponding authors upon reasonable request.
